# Active Women Across the Lifespan: Nutritional Ingredients to Support Health and Wellness

**DOI:** 10.1007/s40279-022-01755-3

**Published:** 2022-09-29

**Authors:** Abbie E. Smith-Ryan, Hannah E. Cabre, Sam R. Moore

**Affiliations:** 1 Applied Physiology Laboratory, Department of Exercise and Sport Science, University of North Carolina, 209 Fetzer Hall, CB#8700, Chapel Hill, NC 27599 USA; 2 Human Movement Science Curriculum, Department of Allied Health Science, University of North Carolina, Chapel Hill, NC USA

## Abstract

Women are the largest consumers of dietary supplements. Dietary supplements can play a role in health and performance, particularly for women. Growing evidence and innovations support the unique physiological and nutrient timing needs for women. Despite the need for more nutrition and exercise-specific research in women, initial data and known physiological differences between sexes related to the brain, respiration, bone, and muscle support new product development and evidence-based education for active women regarding the use of dietary supplements. In this narrative review, we discuss hormonal and metabolic considerations with the potential to impact nutritional recommendations for active women. We propose four potential areas of opportunity for ingredients to help support the health and well-being of active women, including: (1) body composition, (2) energy/fatigue, (3) mental health, and (4) physical health.

## Key Points

**Table:** 

Important sex-based differences exist between biological male and female individuals that may influence nutrition and dietary supplement recommendations.
Hormonal fluctuations throughout the menstrual cycle and with oral contraceptive use result in metabolic alterations that should be considered when making dietary considerations for active women.
Body composition and protein metabolism change throughout a woman’s lifespan and can be supported with sex-specific nutritional recommendations.
There are dietary supplements that target energy, mental health, and physical health for an active woman and may differ from an active man.

## Introduction

With growing participation in sport and exercise amongst women, addressing the nutritional needs of women is critical. Foundational physiological research on women has highlighted sex differences at rest, in response to exercise, and with age [1]. Despite these understood physiological differences between men and women, the representation of women in exercise and nutrition research is severely lacking [2]. In combination with an increasing awareness of the biological sex influences on exercise, women are the largest consumers of dietary supplements with 77% of women utilizing at least one ingredient or supplement [3]. Understanding physiological underpinnings regarding biological sex-based differences between men and women may help optimize nutrient timing, exercise performance, and recovery through supplementation. This narrative review aims to provide an overview of physiological considerations for active women, across the lifespan, which in turn may influence dietary supplement choices to support their needs. This narrative review focuses on biological sex, while also recognizing the impact of female sex hormones on metabolism, body composition, and dietary supplements.

## Physiological Considerations for Active Women

Important sex-based differences exist in metabolism [4], fatigability [5], vasodilation [6], and body composition [7] (for a comprehensive review, see Ansdell et al. [1]). It is well known that most male individuals have a greater quantity of skeletal muscle than female individuals, which often translates to greater strength [1, 8]. However, when physiological properties of skeletal muscles are examined, female individuals tend to have a 7–23% greater proportion of type I muscle fiber types [9, 10], which can impact the capacity for substrate utilization. In terms of metabolism, greater type I muscle fibers oxidize more fat, but less amino acids and carbohydrates, possibly demonstrating increased exercise capacity and reduced skeletal muscle fatigability in women [4, 5]. While trained men display greater glycolytic capacity [11], trained women exhibit faster oxygen uptake kinetics during moderate-intensity exercise and smaller decreases in adenosine triphosphate (ATP) levels and breakdown after all-out exercise compared with male individuals [12–14]. When comparing skeletal muscle mitochondrial oxidative function between trained men and women, women have demonstrated about one-third greater mitochondrial intrinsic respiratory rates [15]. Taken together, female skeletal muscle may be more suited to resynthesize ATP from oxidative phosphorylation during exercise, which has implications for decreased fatigability and increased exercise recovery. Importantly, perfusive and hemodynamic properties of the muscle also vary between sexes. Women have demonstrated greater vasodilatory responses of the arteries, providing more blood flow to exercising skeletal muscle and possibly increasing the delivery of oxygen to and removal of metabolites from the working muscle [6, 12]. Data from muscle biopsies revealed a higher density of capillaries per unit of skeletal muscle in the vastus lateralis among women compared with men [9], which may support greater muscle perfusion during exercise, a key factor in aerobic performance. Elevated estrogen levels in women also promote vasodilation by stimulating nitric oxide (NO) synthesis and decreasing production of vasoconstrictor agents [16]. Given estrogen’s role in substrate utilization and vascular function, changes in estrogen levels across the menstrual cycle may have important implications for exercise capacity in women. Taken together, women appear to be less fatigable compared with men. With a greater proportion of type 1 muscle fibers, dietary supplements that target anaerobic energy production or ingredients that are optimized when higher threshold motor units are activated, such as muscle buffers, may be particularly helpful for active women.

### Hormonal Implications of the Menstrual Cycle

**Fig. 1:**
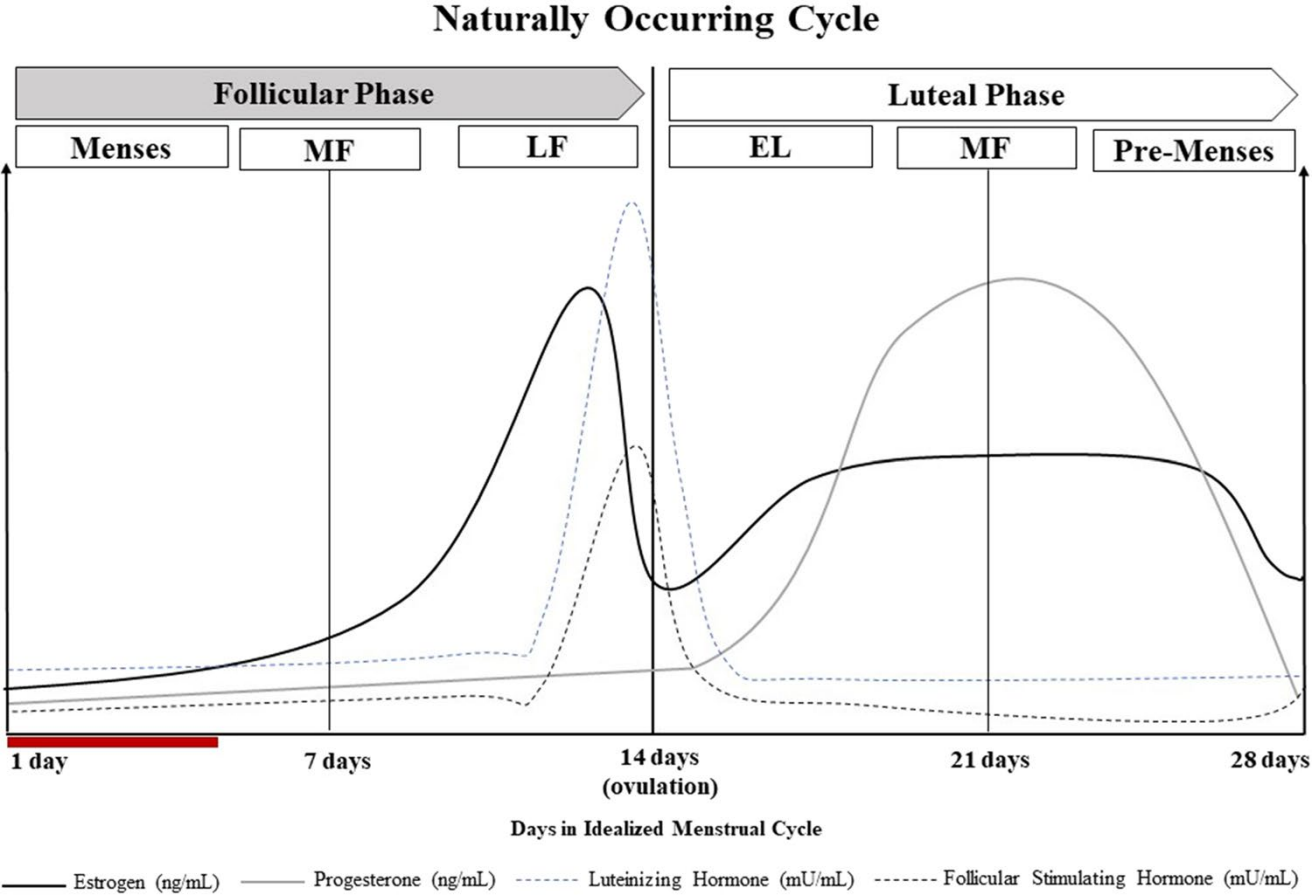
Hormone levels according to an idealized menstrual cycle (28 days). Adapted from Draper et al. [18]. Changing levels of female sex hormones are characterized by the phases of the menstrual cycle. *EL* early luteal, *LF* late follicular, *MF* mid-follicular, *ML* mid-luteal

Between the ages of approximately 12 and 51 years, female individuals experience a circa mensal rhythm termed the menstrual cycle, characterized by predictable fluctuations in ovarian hormones, estrogen and progesterone [17]. A regular menstrual cycle can range from 23 to 38 days (Fig. 1) and consists of three main phases: the follicular phase (FP), ovulation, and the luteal phase (LP). In the beginning of the FP, estrogen and progesterone levels are low (menstrual phase; day 0) and then begin to rise (days 4–5) to prepare the body for ovulation [17, 18]. As the FP ends (days 11–13), estrogen rises and falls with a spike in follicular-stimulating hormone and luteinizing hormones inducing ovulation (day 14). In the second half of the cycle, the LP (day 15), estrogen levels rise again with a concomitant increase in progesterone (days 20–24) to prepare the body for pregnancy. If pregnancy does not occur, estrogen and progesterone levels fall, and the pre-menstrual phase begins [17, 18]. These hormonal fluctuations are an important consideration for eumenorrheic women who exercise as they may influence substrate utilization, performance, and recovery across the cycle.

### Metabolic Considerations Across the Menstrual Cycle

The fluctuations of endogenous hormones across the menstrual cycle facilitate varying modulations in energy expenditure and macronutrient metabolism for eumenorrheic women [19]. Elevated estrogen levels in women also promote vasodilation by stimulating NO synthesis and decreasing production of vasoconstrictor agents [16]. Given estrogen’s role in substrate utilization and vascular function, changes in estrogen levels across the menstrual cycle may have important implications for exercise capacity in women. The implications of the dynamic hormonal landscape as it pertains to fuel utilization and oxidation are of clinical relevance to the development of supplementation and nutritional applications.

#### Follicular Phase Considerations

The FP, as previously described, is characterized by a gradual and substantial rise in estrogen, peaking in the few days prior to ovulation. The gradual onset of estrogen peaking in the late FP provides a much different hormonal environment than the initial hormonal landscape of low estrogen and progesterone [20]. Ovulation presents a unique environment with the greatest estrogen-to-progesterone ratio when estrogen reaches the highest peak of the cycle while progesterone elevates slightly above menstruation levels. While most research does not demonstrate significant differences in resting levels of muscle glycogen, substrate utilization and breakdown across the cycle is well documented [21], suggesting women oxidize less total carbohydrates than men [4]. The impact of low estrogen in the early FP is observed by lower muscle glycogen levels, as a result of increased glucose production, glucose uptake, and carbohydrate oxidation [22]. The increased carbohydrate oxidation results in an upregulation of creatine kinase and interleukin-6 in women, which has been shown to possibly be attenuated with increased carbohydrate consumption during the FP [23]. The reduced capacity for glycogen storage in the early FP has been found to be negated with a carbohydrate-loading protocol of greater than 8 g·kg^−1^·day^−1^ in moderately trained endurance athletes [24]. While the carbohydrate intake recommendations for recreational athletes are far below 8 g·kg^−1^·day^−1^, being mindful of carbohydrate consumption in the early FP can possibly help downregulate heightened inflammatory responses and reduce muscle glycogen utilization.

#### Luteal Phase Considerations

The LP is initiated by ovulation and represented by a post-ovulatory drop in estrogen, signaling the rise and peak of progesterone, the dominant hormone of the LP. Estrogen rises to a second peak, before both decrease to signal endometrial shedding. With progesterone as the predominant hormone, many anti-estrogenic influences can be observed. The increased presence of progesterone promotes the shuttling of glucose into the liver as well as upregulating glucose transporter (GLUT)-4 and GLUT-1 translocation to enhance glycogen storage in the endometrial tissue for embryonic support [25]. As a result, glycogen supercompensation may be more difficult to attain in the LP without higher routine daily carbohydrate levels, owing to changes in insulin sensitivity and a decreased sensitivity of insulin-like growth factor-1-mediated glucose uptake. Therefore, carbohydrate intake during the LP is an important nutrition consideration in order to saturate muscle glycogen for performance, as well as to support the immune system [26]. Globally, energy expenditure is larger in the LP owing to increased demands of cell growth with development of the endometrial lining, indicating an increase in total daily caloric intake may be beneficial to support training loads and performance. As progesterone upregulates to meet increased cell growth demands, a protein biosynthesis requisite for endometrial thickening depletes amino acid levels. Early evidence suggests that consuming more than 1.5 g·kg^−1^·day^−1^, or 0.3–0.5 g·kg^−1^ per meal, may be beneficial to attenuate the increased amino acid oxidation under high-estrogen conditions [27] and could have better implications for recovery.

#### Oral Contraceptives

To date, the majority of available research in women has evaluated naturally occurring menstrual cycles, excluding women who utilize hormonal contraception methods. However, more than 60% of adult women in the USA [28] and 57% of American collegiate woman athletes utilize some form of hormonal contraception [29]. Oral contraceptive (OC) pills are the most common type of hormonal contraception, and the second most common hormonal profile in female individuals [30]. Specifically, exogenous estrogens and progestins in OCs significantly reduce endogenous levels of estrogen and progesterone via chronic down-regulation of the hypothalamic-pituitary-ovarian axis.

Despite the prevalence of OC use in the athletic and general populations, the effects of OCs on exercise performance and metabolism are poorly understood. Furthermore, the differences in endocrine profiles among female individuals (i.e., hormonal contraceptive users and non-users) highlight the need for future hormonal profile considerations within sport and exercise science research. However, understanding the implications of OCs may be helpful in understanding the opportunity for the use of dietary supplements among women.

Combination OCs are the most commonly prescribed type of OC and are found in two different dosage varieties: monophasic and triphasic. Monophasic OC is the prevailing dosage type, characterized by consistent amounts of ethinyl estradiol and progestin throughout the month. Triphasic OCs are distinguished by a consistent dosage of ethinyl estradiol while the synthetic progestin is delivered in low, moderate, and high amounts over 3 weeks, attempting to mimic the gradual onset of progesterone seen in a eumenorrheic menstrual cycle. Modulations in metabolism can be impacted by hormone dosages as well as progestin type and generation (for a full review of progestin generations, see [31]). Oral contraceptive hormone profiles should be considered when evaluating metabolism and performance [32]. Evidence shows OCs with higher dosage amounts can decrease glucose tolerance, in turn augmenting insulin resistance [33], which may be a consideration for carbohydrate and protein timing. An increased C-reactive protein response was also observed through glucose tolerance testing of Olympic female athletes taking OCs, illustrating an upregulated inflammatory response, possibly indicating increased muscle damage or insufficient recovery [34]. Some evidence suggests the impact of monophasic OC use in exercising women is more indicative of increased triglyceride mobilization and plasma cortisol levels when compared to recent carbohydrate consumption or endogenous hormones of the menstrual cycle [35]. Early data demonstrate significantly higher oxidative stress in female athletes using OCs than their non-OC user counterparts irrespective of lifestyle habits [36]. The evaluation of the impact of OCs on performance is far behind where it needs to be. Based on existing physiological data, dietary supplements may help to modulate the inflammatory response, reduce muscle damage, and possibly lower oxidative stress among those utilizing OCs. Scarce data also indicate that weighing the benefit-to-risk ratio of dietary supplements for these outcomes among women using OCs will be important.

## Body Composition

Understanding sex-specific characteristics of body composition is important for optimizing performance, recovery, and overall health [7]. In general, men have more lean mass (LM), while women have more fat mass (FM) and a higher body fat percentage; men are more susceptible to gaining abdominal fat (android), whereas women usually carry more fat in the hip (gynoid) region [7]. Nutritional support and modifications can help to enhance optimal lean mass and body composition for active women, and this may differ between sexes.

### Body Composition Changes Across the Female Lifespan

**Fig. 2:**
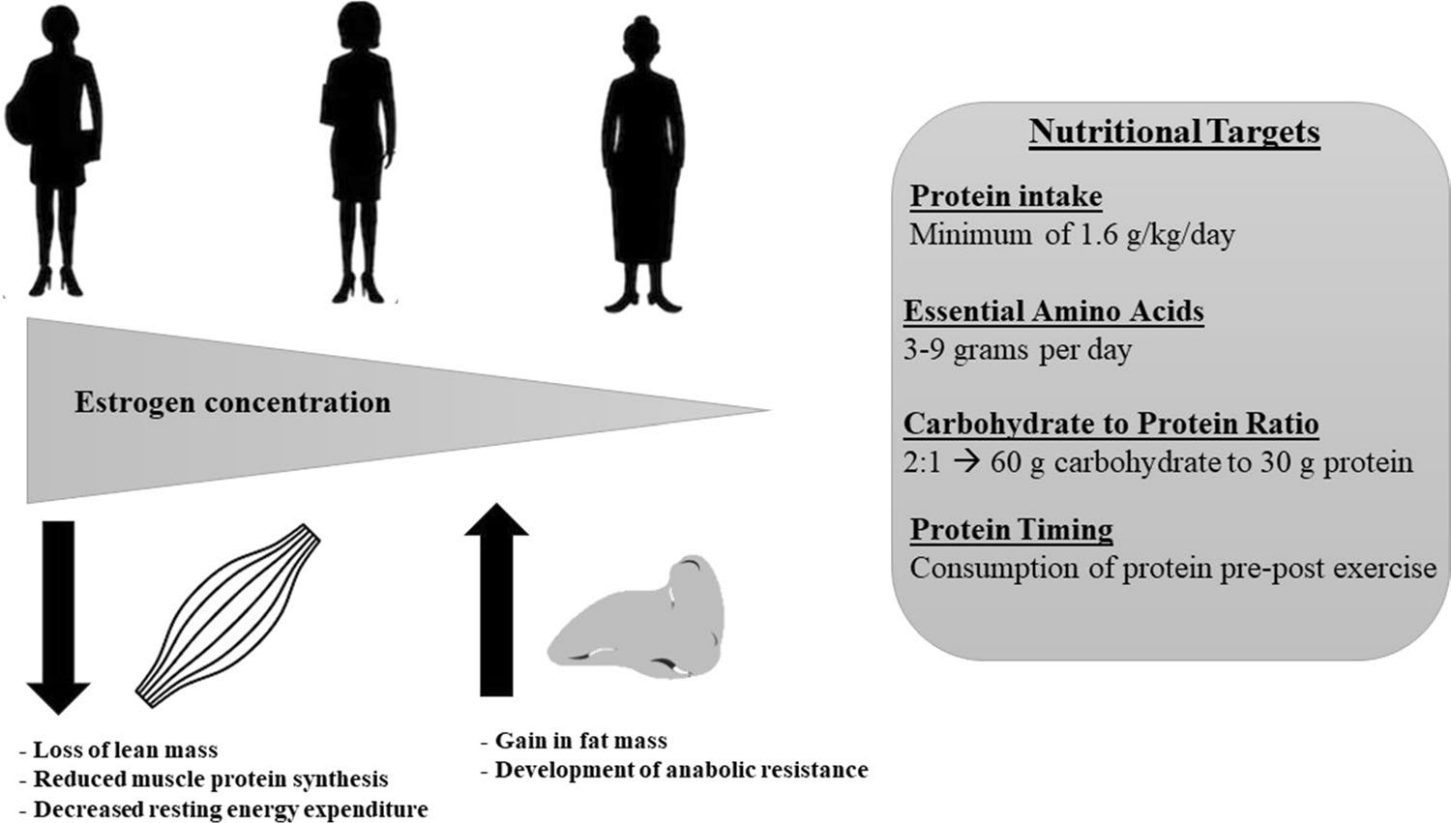
Key considerations in body composition across a woman’s lifespan and nutritional considerations to address body composition changes

Sex-dependent differences in body composition begin at puberty primarily due to estrogen’s role in the regulation of body composition in female individuals. Estrogen receptors are widely distributed across tissues such as adipose tissue and skeletal muscles [37]. Their involvement in genomic and non-genomic signaling pathways demonstrates the potential role of estrogen in body weight regulation and other metabolic processes (Fig. 2) [38]. Estrogen levels remain consistent from puberty into adulthood. However, in the years preceding menopause, known as peri-menopause (average age 45 years), estrogen levels begin to dramatically decrease until the final menstrual period, known as menopause (average age 51 years) [39]. Changes in energy expenditure across the female lifespan hold important implications for body composition. Estrogen receptors are located on the mitochondria, suggesting estrogen signaling may mediate the regulation of body composition and energy balance (i.e., energy expenditure and intake) [38].

Estrogen is regarded as a master regulator of bioenergetic systems in the female body. When resting energy expenditure was measured in pre-menopausal women during the mid-luteal phase of the menstrual cycle (elevated estrogen and progesterone), the early follicular phase (low estrogen), and after 6 days of estrogen suppression, the changes in resting energy expenditure paralleled the changes in estrogen. Resting energy expenditure was the highest in the mid-luteal phase, lower (− 29 kcal/day) in the early follicular phase, and further reduced (− 42 kcal/day) after estrogen suppression [40]. These results suggest elevated estrogen promotes energy expenditure in female individuals and that resting energy expenditure may vary across the menstrual cycle. Therefore, menstrual cycle phase and level of estrogen may be key considerations when choosing supplementation to support body composition changes. As women transition from peri-menopause to post-menopause, the loss of estrogen may decrease energy expenditure. In a 1-year longitudinal study, 24-h energy expenditure decreased significantly with age and fat oxidation decreased by 32% in women who became post-menopausal compared with premenopausal women [41]. There is also consistent evidence from basic and preclinical research that the disruption of estrogen signaling accelerates fat accumulation, and the loss of estrogen at menopause is likely to have pronounced effects on body composition [38]. Data from the longitudinal Study of Women’s Health Across the Nation (SWAN) cohort demonstrated the rate of fat gain in pre-menopausal female individuals was an average of 0.25 kg per year, but over the ~ 3.5-year menopause transition, adverse changes in body composition began to accelerate [42]. The rate of fat gain doubled to 0.45 kg per year leading to a 6% total gain in FM (an average absolute gain of 1.6 kg) and an average 0.5% loss in LM [42]. These unfavorable alterations in body composition, which abruptly worsen at the onset of the menopause transition, indicate peri-menopause may be a key time period for exercise and nutritional interventions [43], particularly in regard to maintenance of LM, mitigating FM gain, and alterations in energy expenditure.

### Skeletal Muscle Mass Considerations

At rest in a basal fasted state, there appear to be few differences in muscle protein synthesis (MPS) between younger male and female individuals, but the rate of MPS may be dependent upon LM content [44]. The hormonal differences observed throughout the menstrual cycle have been proposed to have some influences on protein kinetics. In regard to myofibrillar or connective tissue proteins, there appear to be no differences in basal MPS in pre-menopausal female individuals between the menstrual cycle phases at rest, despite variations in estrogen and progesterone [45]. However, some studies suggest there are small changes in protein kinetics during the LP, with greater utilization of protein at rest and with exercise [18, 46]. Interestingly, the use of OCs may also lower basal MPS [47], but it does not appear that long-term OC use has a markedly negative influence on the adaptive responses to exercise in terms of performance [48], although this is largely unexplored. Initial evidence in young female OC (ethinyl estradiol) users demonstrated greater improvements in muscle hypertrophy with a 10-week progressive resistance training intervention, but no differences in strength compared to non-OC users [49]. In contrast, in post-menopausal women, estradiol had no impact on MPS [50]. Because of hormonal profile differences between eumenorrheic women and women using hormonal contraceptives, as well as women transitioning to menopause, this would be an important area for future research in MPS.

In the transition from pre-menopause to peri-menopause, preliminary data indicate a pronounced decrease in skeletal muscle protein balance [43]. Menopausal-related hormonal changes and losses in muscle mass may be a direct result of skeletal muscle dysregulation owing to the development of anabolic resistance to nutrient intake, particularly to the precursor essential amino acids (EAAs) [43, 51–53]. The reduced capacity for protein anabolism leads to a decrease in protein turnover and, eventually, a loss of muscle size and quality, particularly in post-menopause. These changes in protein kinetics may contribute to the adverse body composition changes observed during the menopause transition. The process of protein turnover, particularly protein synthesis, is energetically expensive, indicating the rate of protein turnover may be a primary determinant of total daily energy expenditure [54]. As such, decreases in protein turnover can significantly influence energy balance. Additionally, because the energy required for protein turnover is primarily derived from fatty acid oxidation, a reduction in protein turnover corresponds with a reduction in fatty acid oxidation and potential subsequent accumulation in fat mass, contributing to the body composition changes observed during the menopause transition. With greater anabolic resistance, downregulated MPS, and potential decreased fatty acid oxidation, supplement strategies that target these alterations may be particularly important for active women, and women as they age.

### Protein Supplementation Considerations

For the active woman, similar to an active man, stimulating MPS is a key factor for the adaptive responses to exercise with implications for improvements in body composition, particularly LM. The consumption of adequate dietary protein is essential to support exercise training adaptations particularly the remodeling of protein structures, augmentation of LM, and strength [55, 56]. Because of the increased protein turnover associated with exercise, current sports nutrition guidelines for daily protein intake are 1.2–2.0 g/kg/day to optimize exercise training adaptations [56, 57]. In female athletes specifically, very limited research has been conducted on the protein requirements, but existing data suggest that active women should consume a minimum of 1.6 g/kg/day of protein [58]. Higher protein diets (> 2.0 g/kg/day) have been shown to be important for maintaining LM and resting energy expenditure under periods of intentional and unintentional caloric restriction [59], which may be more prevalent among active women. Additionally, high-protein diets (> 2.2 g/kg/day) have not resulted in any adverse effects to bone mineral density or kidney function in healthy women after 6 months [60] or 1 year [61]. As a result of the potentially greater protein oxidation, particularly in the LP, active women should focus on consuming high-quality protein sources (e.g., high biological value, high digestibility, absorption, and EAA content) to minimize muscle soreness and breakdown.

The loss of LM is a common characteristic of age; women appear to experience a greater loss of LM with age compared with men [62]. Early evidence suggests there may be a reduction in whole-body protein turnover as women transition to menopause [63], as well as a reduced anabolic response to amino acid intake in post-menopausal women, compared with men [64]. This blunted sensitivity appears to be overcome when larger amounts of the EAAs including leucine are ingested, suggesting an EAA supplement may be an important intervention to mitigate LM loss with age [51, 53]. When paired with exercise, amino acids synergistically influence rates of MPS through activating the mechanistic target of rapamycin complex 1 signaling pathway [52, 65]. A study demonstrating consumption of bolus whey protein (20 g) versus low-dose leucine-enriched EAAs (3 g) in older women demonstrated both protein types equally stimulated MPS 0–2 h after ingestion. Additionally, when the two protein supplement types were paired with exercise, MPS increased at 0–2 h and remained elevated at 0–4 h for both dietary supplements [66]. These results suggest supplemental intake of EAAs may be an equally beneficial supplementation compared with whey protein for middle-aged and older female individuals. This is of particular importance as the lower dose may be more feasible for consumption. These data also highlight the potential benefit of a supplemental form of protein to optimize absorption to maximize MPS.

**Fig. 3:**
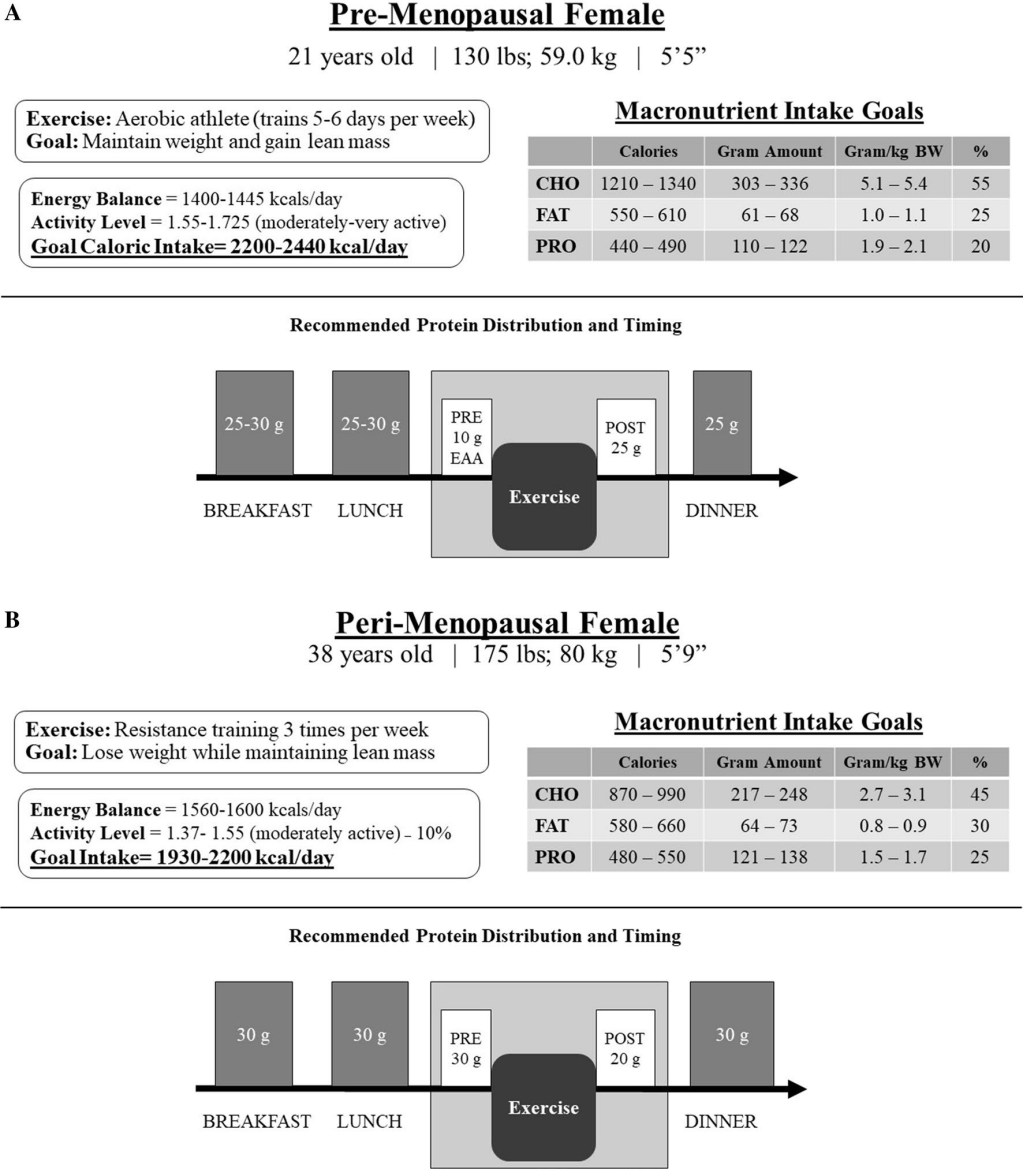
Energy needs and protein timing considerations for **A** pre-menopausal and **B** post-menopausal active women. Total daily calorie estimations were based on the Harris-Benedict equation and relevant activity factor based on activity level. For the peri-menopausal woman, her calorie intake has been reduced by 10% to ultimately reach a hypocaloric intake for a weight loss goal. *kg* kilograms, *BW* body weight, *lightly active* light exercise/sports 1–3 days/week, *moderately active* moderate exercise/sports 3–5 days/week, *very active* hard exercise/sports 6–7 days a week

Protein supplementation through whey protein sources or EAAs can complement high-quality proteins consumed through the diet. When considering the distribution of macronutrients, the carbohydrate-to-protein ratio has been shown to be important for supporting optimal body composition and fat loss in women. The consumption of a ratio of 2:1 carbohydrate to protein has demonstrated significant losses in percent body fat while supporting a gain in LM [67–69]. This ratio supports a higher protein intake while supporting lower carbohydrate needs to optimize fat oxidation. Female individuals may be especially sensitive to improvements in the carbohydrate-to-protein ratio for FM/LM alterations, suggesting that macronutrient distribution in addition to increased protein intake may be important for body composition changes. A practical example of how protein timing and intake might be distributed, while also considering total daily energy expenditure, macronutrients, and goals, is displayed in Fig. 3. Additionally, research on the timing of nutrients around exercise in active women has highlighted the importance of amino acid availability in regulating MPS, metabolism, and adaptations to exercise [70, 71]. At rest, basal rates of MPS and fat oxidation are blunted when fasted compared with postprandial, particularly in women [72]. When comparing acute consumption of protein (90 kcals) compared with carbohydrate (90 kcals) prior to exercise (interval training vs resistance training vs aerobic exercise), protein consumption resulted in greater energy expenditure and fat oxidation after exercise [73]. Another study observing both pre-exercise and post-exercise protein consumption demonstrated increases in maximal upper body strength in female individuals during resistance training, with greater effects resulting from pre-exercise consumption [71]. In female endurance athletes, protein intake consumed immediately post-exercise improved nitrogen balance compared with protein intake consumed in the morning [74]. Taken together, these data indicate that consuming protein either before or after exercise is a key factor in MPS in active women, and the timing of protein may be even more critical for women compared with men. Because of the potential for gastrointestinal disturbances, a dietary supplemental form of protein/EAAs may be important to support these physiological benefits. Future research should consider the use of protein supplementation, particularly EAAs, across the phases of menstrual cycle, which has yet to be explored.

## Supplementation for Energy and Fatigue

The most common reason women cite for use of dietary supplements is increased energy [3]. Considering the lifestyle of active women, which often includes reported levels of multi-tasking, invisible labor, child rearing, and a career, an optimizing formulation and use of dietary supplements may be warranted (Table 1). Physiologically, sleep is a key consideration, but this topic will be left for a more behavioral discussion. While there is a need for more female-specific dietary supplement research, based on available science, there are a few ingredients that may provide advantages for active women with the desire to delay fatigue and increase energy around exercise and in day-to-day life. When evaluating a dietary supplement, third-party testing is imperative to verify product contents and rule out contaminants. The most common and recommended certification programs to look for when identifying evidence-based supplements include: National Sanitation Foundation, National Sanitation Foundation for Sport, Informed Choice, Consumer Labs, and the Banned Substances Control Group. The US Pharmacopeia also provides third-party verification for vitamins.

**Table 1:**
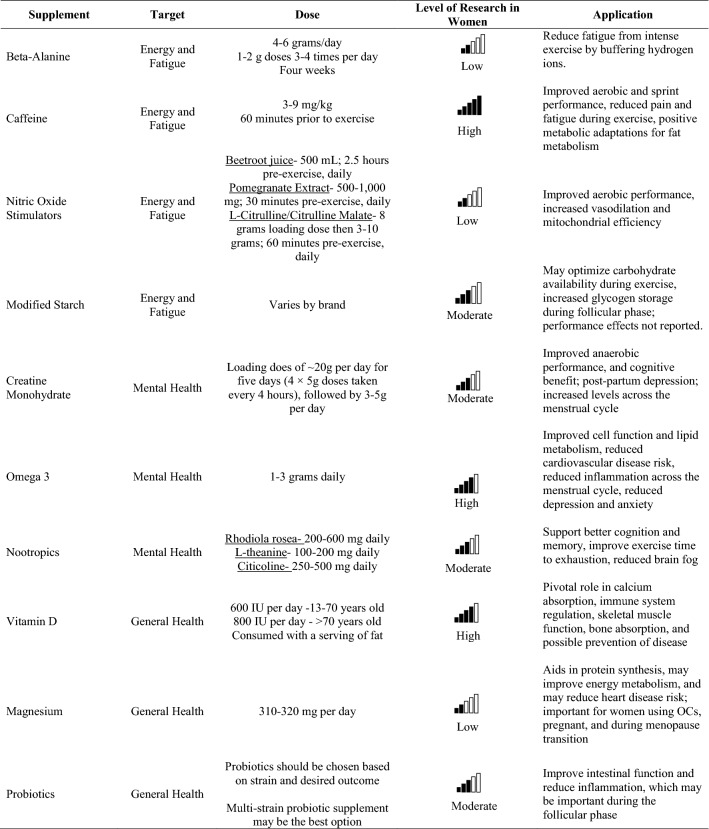
Supplement considerations for the active female individual

*OCs* oral contraceptives

The level of research evaluation included contributions from available data completed in women, sample size of research in women, consistency of findings across studies, and strength of impact on listed outcomes

### Beta-Alanine

Beta-alanine is a non-EAA that has been shown to delay fatigue, particularly in activities lasting 2–4 min. Active women who are beginning a new exercise routine or having difficulties recovering from higher intensity shorter duration exercise may benefit from beta-alanine supplementation. Beta-alanine is the rate-limiting substrate for carnosine synthesis, an innate hydrogen ion buffer. When carnosine levels are elevated as a result of beta-alanine supplementation, fatigue has been delayed during exercise [75]. Individuals who have low muscle carnosine levels, such as women, older adults, and vegetarians, could see greater benefits from beta-alanine supplementation [76], especially when participating in high-intensity exercise [77]. As a result of the loss of LM with age and subsequent decrease in muscle carnosine levels, older active women may realize even greater benefits from supplementation; middle-aged women (54 years) demonstrated significant improvements in lower body knee extension strength following 28 days of beta-alanine supplementation [78]. A combined sample of older men and women (aged 55–92 years) also demonstrated significant improvements in fatigue resistance [79]. Supplementation with beta-alanine requires a loading phase of ~ 28 days in order to maximize muscle carnosine levels. To date, it does not appear to differ between men and women. A total dose of 4–6 g/day divided into 1–2 g doses for 4 weeks is recommended to significantly augment muscle carnosine levels. There are additional data that suggest beta-alanine may have some antioxidant properties, [80] which may be relevant for women during the FP of the menstrual cycle to support better recovery, as well as data to support better heart rate recovery [81]. With a 14-week half-life, beta-alanine is not something that needs to be taken all the time, but instead cycled on and off as needed.

### Caffeine/Teacrine

Caffeine is one of the mostly widely studied and used ingredients, acting as a central nervous system stimulant. There are physiological considerations for female-specific caffeine recommendations. Caffeine elimination appears to fluctuate over the course of the menstrual cycle with slower elimination and more pronounced effects during the LP as well as with OC use [82]. The accumulation of caffeine during this high-estrogen phase may magnify premenstrual symptoms, as well as intensify the sympathetic effects of caffeine, resulting in increased heart rate, anxiety, and impaired sleep [83–85]. Caffeine is effective for improving repeated sprint/intermittent exercise performance and endurance performance because of its ability to reduce pain perception and augment fat oxidation, thereby sparing muscle glycogen [83]. The effects of caffeine appear to be similar between men and women from a 3-mg/kg dose in an aerobic exercise setting, although women demonstrated a greater plasma caffeine level with the same dose [86]. It should be noted that studies to date evaluating the effects of caffeine in women have not accounted for the menstrual cycle. Additionally, if a woman consumes caffeine habitually, a higher dose (6 mg/kg) may be needed to see significant improvements in anaerobic performance [87]. A typical recommended dose of 3–6 mg/kg consumed 60 min prior to exercise can delay fatigue/increase energy. Teacrine is a naturally occurring purine alkaloid that is similar to caffeine. It is most commonly found in tea and coffee, and acts as an adenosine receptor antagonist, as well as supporting a positive benefit on mood and energy [88], without negatively affecting tolerance. The half-life of teacrine is 2 h and it has been shown to be safe and effective for delaying fatigue, both physically and cognitively [89]. The combined effects of caffeine and teacrine may be a unique combinatory approach for increasing energy and delaying fatigue in women, but more female-specific research with teacrine is warranted.

### Nitrates

Dietary nitrate supplementation has attracted substantial interest over the past decade for its role in health and athletic performance. Nitrate products are thought to increase NO production through the NO synthase-dependent pathway of NO production, which includes a series of reactions oxidizing l-arginine to l-citrulline and NO. Specifically, NO is a potent signaling molecule that elicits changes in biological and physiological processes such as vasodilation, mitochondrial efficiency, and calcium handling, all of which have important implications for exercise capacity [90]. Sex-based differences in physiology and biological processes may influence NO production [91]. Compared with male individuals, female individuals have higher baseline NO levels and may have greater increases in NO following nitrate supplementation, although this is largely influenced by dosing [91]. Additionally, the effects of nitrate supplementation appear to be more effective in early post-menopausal women for supporting improvements in blood pressure [92], compared with premenopausal women, which highlights the impact of estrogen on endothelial tissues. Women have demonstrated increased blood flow during intermittent exercise. However, female individuals have smaller vessels indicating they may be more likely to benefit from nitrate intake particularly as it relates to vasodilation. Female individuals also have a greater ability to reduce nitrates to NO compared with male individuals, suggesting nitrate supplementation may be more effective in women compared with men [91]. Supplementation with nitrates may be particularly important for aerobic activities and for delaying fatigue during exercise, as well as in women as they age. More recently, acute and chronic supplementation with beetroot juice (280 mL/day) in young female OC users did not improve aerobic performance, but did improve torque production [93]. Additionally, 140 mL of beetroot juice consumed 2.5 h prior to exercise in trained female individuals resulted in no effect on oxygen consumption but did reduce perceived exertion [93]. The impact of dietary nitrate supplementation on recovery or across the menstrual cycle has not yet been explored in women. Dietary nitrates are most commonly found in green leafy vegetable and root vegetables, and dietary supplementation forms of nitrates include beetroot juice and pomegranate extract as well as citrulline and arginine. Dosing and timing recommendations are provided in Table 1 [94].

### Carbohydrates and Modified Starch

The importance of carbohydrate availability for exercise performance is well established [95]. As a result of sex-based differences that exist in carbohydrate and fat oxidation during exercise [22], as well as differing sensitivities of the gastrointestinal tract among active women [96], carbohydrate supplementation during exercise is ergogenic [95]. As noted in Sect. 2.2, glycogen utilization and storage changes throughout the menstrual cycle, which may impact carbohydrate feeding recommendations, particularly throughout exercise. Increasing the amount of carbohydrate intake (8–12 g/kg/day) for women to sufficiently load the muscle with glycogen may be necessary when supercompensation is warranted. The intricacies and practicalities of this are previously described [19]. In concert with carbohydrate feeding during exercise, symptoms of gastrointestinal distress have been reported to be more prevalent in female endurance athletes, and among those women consuming hypotonic beverages [97]. Other forms of carbohydrate supplementation may be important to consider for active women, particularly for women undergoing endurance exercise. Modified starches may affect the gastric-emptying rate, enhancing glycogen storage, [98] which may be beneficial during the FP, or to spare glycogen by enhancing fat oxidation, which may also be beneficial as estrogen and progesterone levels change. To date, research has failed to demonstrate a positive effect of a fast-digesting high-molecular-weight starch in female cyclists [99] or a slow-digesting modified starch [98]. There are some potential positive data for modified starch on performance, but only in men. This area needs additional study but addressing and modifying the carbohydrate source may be helpful for the active woman undergoing exercise activities that rely on muscle glycogen, as well as to mitigate gastrointestinal distress.

## Recommendations for General Health

### Creatine

The benefits of creatine supplementation for women are growing in evidence, with positive benefits related to strength, hypertrophy, performance, as well as energetic and cognitive outcomes [100]. Changes in endogenous hormone levels may underpin varying creatine characteristics between male and female individuals, with female individuals demonstrating 70–80% lower endogenous stores and consuming considerably lower amounts of dietary creatine [101]. Creatine kinase modulations have also been shown to align with the cyclical pattern of estrogen across the menstrual cycle [102]. Fluctuations in creatine kinase levels have been reported to be influenced by endogenous hormones, with the lowest levels observed during non-menstruating years, and subsequent decreases with age and pregnancy [103]. Creatine supplementation may be particularly effective post-partum as a result of cellular energy depletion surrounding childbirth [104]. Decreases in muscle mass, bone mass, and muscle strength, resulting from decreased estrogen levels observed during the menopause transition, have also shown to be attenuated with creatine supplementation [105, 106].

Short-term and long-term creatine supplementation have shown significant beneficial ergogenic outcomes in strength, hypertrophy, and exercise performance in trained and untrained female populations when compared with placebo controls [107]. Mechanisms supporting increases in strength, hypertrophy, and performance are likely related to increased intramuscular phosphocreatine stores, which allow for a greater stimulus of training through greater energy availability from increased ATP turnover during exercise. Delayed neuromuscular fatigue allows for improved recovery and prevention of fatigue through maintenance of pH [108]. Data also suggest positive relationships between mood and severity of depressive episodes with creatine and phosphocreatine levels in the brain [109]. Research has demonstrated a reduced time for acclimatization of anti-depressant medications for expedited effectiveness with creatine supplementation [110]. Creatine supplementation has also shown to effectively reduce mental fatigue and improve cognitive performance, specifically during times of high duress or impacted sleep quantity or quality [111]. A common misconception surrounding creatine supplementation pertains to undesirable weight gain in women; however, research shows initial gains incurred with loading doses are likely a result of increased cellular hydration (i.e., “water weight”), which can show favorable outcomes for hydration [112]. Currently, evidence shows consistent recommended dosage amounts for male and female individuals. Creatine supplementation typically follows a pattern of a loading does of ~ 20 g per day for 5 days (4 × 5-g doses taken every 4 h), followed by 3–5 g per day [113].

### Essential Fatty Acids

Our essential fatty acids, omega 6 and omega 3, are key modulators of cell function, lipid-soluble vitamin absorption, and lipid metabolism. Irrefutable evidence has demonstrated a significant reduction in the risk of cardiovascular disease with an increase in omega 3 fatty acids [114]. This is particularly relevant for women, as the risk of cardiovascular disease is exacerbated as they transition to menopause. The two most active eicosanoids derived from omega 3 are eicosapentaenoic acid and docosahexaenoic acid, which play a vital role in improving immune function and aid in growth and development [115]. Existing literature has demonstrated improved inflammatory environments following omega 3 supplementation, including arthritis, inflammatory bowel disease, and asthma. This may be particularly beneficial during the FP of the menstrual cycle when systemic inflammation is elevated. Essential fats are also needed to prevent and counteract relative energy deficiency syndrome [116]. Other potential benefits from elevated omega 3 intake include a decrease in muscle soreness as a result of reducing inflammation [117], increased muscle protein synthesis by stimulating the mechanistic target of rapamycin complex 1 pathway [118], and improved bone health [119]. For women who demonstrate greater anabolic resistance [120], this could be efficacious. Additionally, increased levels of omega 3 have been shown to reduce symptoms of depression and anxiety [121], which are reported in higher rates in women versus men. Benefits of omega 3 have been reported when 1–3 g daily are consumed [122, 123].

### Nootropics

There is a growing category of dietary supplements, ‘nootropics,’ that target cognition and memory, with some also supporting better exercise outcomes. Nootropics have gained even more traction among active women during the coronavirus disease 2019 pandemic. Data report greater levels of stress, sleep, and mental impairments among women during the pandemic [124], with other reports describing a cognitive-like ‘fog’ [125]. With women participating in greater more effective multi-tasking and invisible work [126], nootropics may support better cognition and memory. There are a few that have been substantiated, including rhodiola, l-theanine, ashwagandha [127, 128], *Cordyceps* [129], choline/citicoline [130], and *Bacopa monnieri* [131]. While this is not meant to be an exhaustive literature review, the following ingredients may be beneficial to support cognition in the active woman. *Rhodiola rosea* has been shown to reduce fatigue, improve exercise time to exhaustion, as well as improve mood with 200–600 mg daily [132, 133]. Some of the research is mixed using *Rhodiola*, which is likely attributed to the varying integrity of rosavins. L-theanine is a non-proteinic amino acid that can cross the blood–brain barrier, resulting in improved attention, particularly with individuals with reported anxiety [134]. L-theanine is often combined with caffeine; positive effects have been demonstrated with 100–200 mg dosages. Citicoline has demonstrated cognitive-enhancing and neuroprotective properties in pre-clinical and clinical studies. Following 28 days of citicoline supplementation (250 mg and 500 mg) in middle-aged women, there was a significant improvement in attention [130]. Including a nootropic ingredient when formulating or choosing dietary supplements for active women is supported by increasing evidence, particularly if they are experiencing sleep deprivation, anxiety, and brain fog.

### Vitamin D

Vitamin D is traditionally known for its pivotal role in calcium absorption. However, it is also imperative for innate and acquired immune system regulation, skeletal muscle function, bone absorption, and possible prevention of disease [135]. Research suggests that as women age, vitamin D levels decrease with the highest rate of vitamin D deficiency occurring in post-menopausal women [136]. Low vitamin D in women is linked with a myriad of health consequences such as an increased risk of heart disease, acute illness, stress fractures, muscle pain/weakness, and inflammatory injuries. Vitamin D deficiency is defined as having < 75 nmol/L of circulating 25-hydroxyvitamin D or < 30 nmol/L of vitamin D [137]. The recommended dietary allowance for vitamin D is 600 IU per day for female individuals aged 13–70 years and 800 IU per day for female individuals aged > 70 years [137]. For active women, vitamin D levels could directly affect muscle strength and performance, recovery from exercise, and bone health. Furthermore, vitamin D deficiency has been shown to increase the risk of anemia, which is highly prevalent among active women [138]. Therefore, vitamin D supplementation is recommended for women across the lifespan and across all activity levels. Additionally, a focus on dietary vitamin D through foods such as fish, cheese, and some cereals is an important consideration for women [137]. Vitamin D is fat soluble, which means it needs to be consumed with at least one serving of fat.

### Magnesium

**Fig. 4:**
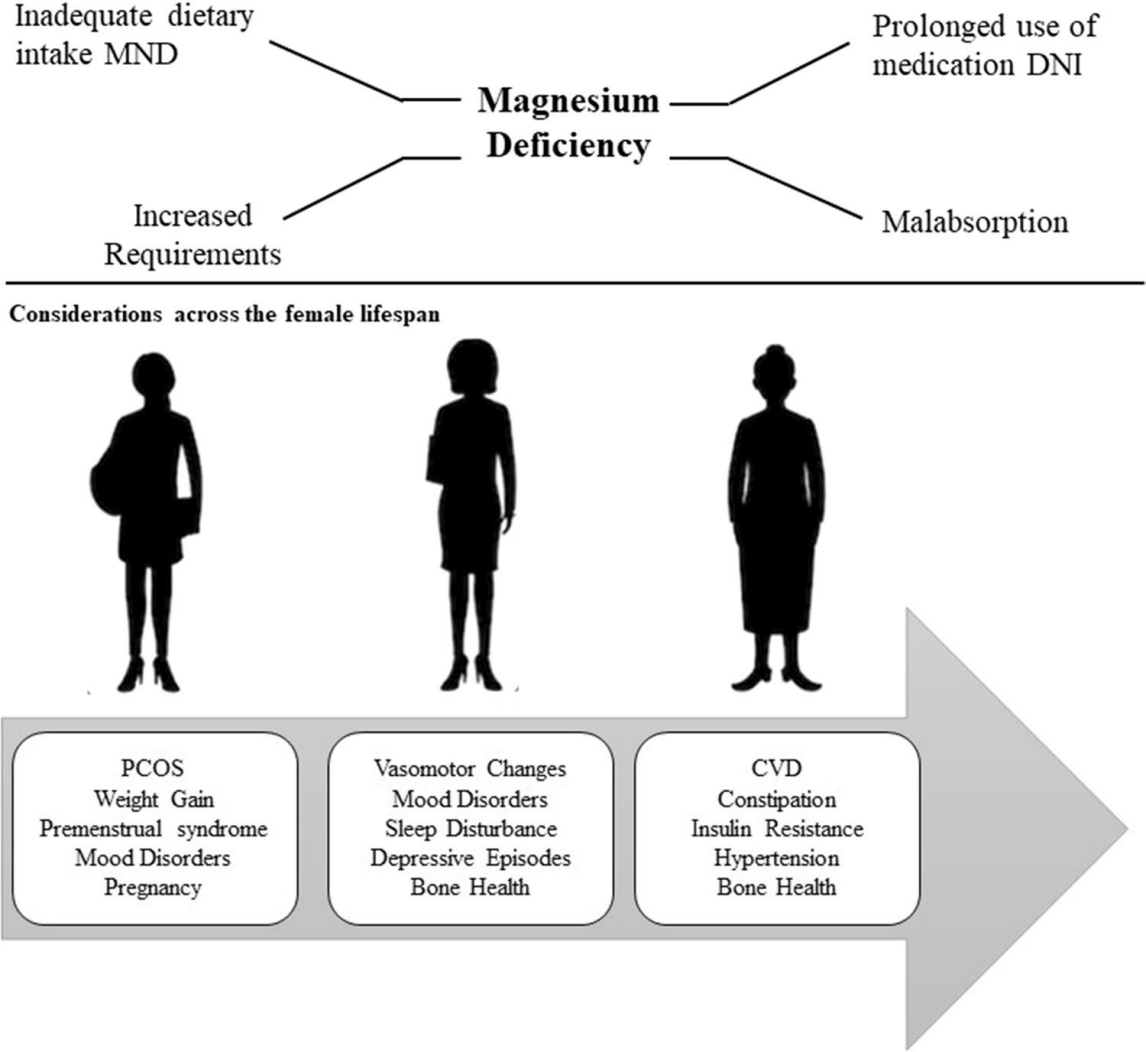
Considerations for magnesium intake across the female lifespan and for factors influencing magnesium deficiency. *MND* micronutrient deficiency, *DNI* drug nutrient interactions, *PCOS* polycystic ovary syndrome, *CVD* = cardiovascular disease

Minerals, such as magnesium, are essential inorganic elements necessary for most metabolic processes. Magnesium activates enzymes involved in protein synthesis, is involved in ATP reactions, and may improve energy metabolism [139]. Acute changes in plasma magnesium levels are noticeable during a continuous bout of moderate-to-high intensity exercise [94]. There is growing evidence to support an essential role of magnesium in various physiological outcomes for women as they age [140]. Normal serum magnesium levels range between 0.75 and 0.95 mmol/L, and magnesium deficiency consists of magnesium levels < 0.75 mmol/L. Overall, magnesium deficiency in healthy individuals consuming a balanced diet is rare, but dosing requirements may change in response to hormonal variations and training adaptations. Specifically, there are various pathophysiological conditions across the female lifespan, such as use of OCs, pregnancy, and menopause, that may increase magnesium requirements (Fig. 4) [140]. Magnesium supplementation in pre-menopausal women may improve premenstrual syndrome symptoms through decreasing inflammatory markers [141]. In peri-menopausal to post-menopausal women, magnesium supplementation may be protective for bone health through optimization of the vitamin D status [142]. Additionally, as women traverse through menopause, there is an increased risk for hypertension. Emerging data suggest magnesium has an inverse relationship with hypertension risk, suggesting magnesium supplementation may have cardioprotective benefits [140], especially for women. The recommended dietary allowance for magnesium in women is 310–320 mg per day, but magnesium supplementation may be needed if the recommended dietary allowance is not met through the diet alone. Magnesium-rich foods include nuts, almonds, bananas, black beans, brown rice, cashews, spinach, seeds, and whole grains (Table 2).

### Probiotics

The gastrointestinal tract of a woman begins to differ from a man at the onset of puberty and continues to change with hormonal fluctuations [143]. Early evidence suggests that women have lower intestinal permeability and higher microbial diversity but are more sensitive to perturbation [144]. Women also have reported greater symptoms of irritable bowel syndrome and symptoms of leaky gut, particularly with exercise. Probiotics have been shown to be an effective stimulus for promoting bacterial diversity and targeting many aspects of health [122, 123]. Probiotic supplementation has been shown to improve intestinal function and reduce inflammation, which may be effective for modulating changes in inflammation during the FP. Probiotics in women have also resulted in improved absorption of iron, when combined with iron supplementation, which could help to reduce iron-deficient anemia, as well as enhance the absorption of some amino acids, which may enhance MPS [145, 146]. Evidence also suggests probiotic use in women may effectively reduce the reoccurrence of urinary tract infections [122, 147]. Probiotics should be chosen based on strain and desired outcome; female-specific probiotics should be further developed to support a woman’s microbiota and symptoms. A multi-strain probiotic supplement is likely the most feasible approach to see the most benefit.

## Conclusions

Women have unique and changeable hormone profiles that influence their physiology and nutritional needs. Understanding the menstrual cycle, OC use, and the ever-evolving hormonal landscape as women age is essential in order to support a woman’s health and well-being as she ages and desires to remain active. Evidence supports the use of female-specific ingredients to optimize body composition, delay fatigue, and improve mental and physical health. Future research and product development must include women across the lifespan and begin to expand upon their needs to improve health, quality of life, and performance.
